# Autonomous Reading of Gauges in Unstructured Environments

**DOI:** 10.3390/s22176681

**Published:** 2022-09-03

**Authors:** Edoardo Milana, Oscar Hernán Ramírez-Agudelo, Jacob Estevam Schmiedt

**Affiliations:** Institute for the Protection of Terrestrial Infrastructures, German Aerospace Center (DLR), 53757 Sankt Augustin, Germany

**Keywords:** analogue gauges, detection systems, computer vision, infrastructures protections, autonomous measurements

## Abstract

This paper introduces GAUREAD, an end-to-end computer vision system that is able to autonomously read analogic gauges with circular shapes and linear scales in unstructured environments. Existing gauge reading software still relies on some manual entry, like the gauge location and the gauge scale, or they are able to work just with a frontal view. On the contrary, GAUREAD comprises all the necessary steps to make the measurement unconstrained from previous information, including gauge detection from scene, perspective rectification and scale reconstruction. Our algorithm achieves a speed of 800 milliseconds per reading on the NVIDIA Jetson Nano 4 GB. Experimental tests show that GAUREAD can provide a measurement with an error within 3% for perspective angles below 20° and within 9% up to 50°. The system is foreseen to be implemented on mobile robotics to automatise not only safety routines, but also critical security operations.

## 1. Introduction

Thanks to the extraordinary advances in the fields of Robotics and Computer Vision, automating security and safety processes in real-time has become a concrete option [1]. Many industrial environments still rely on analogical sensors and gauges to monitor processes and conditions of infrastructures and often there is no possibility to have remote checks. Indeed, periodical inspections still require human labour, with all the safety risks that might occur to workers during the inspections, as well as the time-consumption and high costs.

Therefore, deploying autonomous agents such as drones and wheeled robots is a promising solution. Moreover, recent commercialization of legged robots (Spot from Boston Dynamics [2] and ANYmal from ANYbotics [3]) make them a great alternative, in particular for operations in environments with uneven terrains. Those agents can be equipped with gauge reading algorithms using on-board cameras and computational units.

State-of-the-art algorithms that read analogical gauges make use of Circle Hough Transform to detect and calibrate the gauge display and classic Line Hough Transform to detect the needle position [4]. The needle detection can be done either on the Cartesian reference of the image or on its polar transformation (following the calibration of gauge display). Indeed, the gauge value reading consists of calculating the needle angle with respect to the polar coordinate reference of the calibrated gauge display. Assuming that the gauge scale is linear and knowing the minimum and maximum scale values ($v_{min}$ and $v_{max}$) and their angles on the polar representation ($\theta_{min}$ and $\theta_{max}$), the needle angle ($\theta_{needle}$) can be easily converted in the gauge value using the following:

(1) $$v_{gauge}=v_{min}+\frac{\theta_{needle}-\theta_{min}}{\theta_{max}-\theta_{min}}\left(v_{max}-v_{min}\right)$$

In most algorithms, the polar angles of the minimum and maximum scale values are determined in advance with a manual calibration, so that they are readily available to compute the gauge value. Alegria and Serra [5] used Linear Hough Transform to detect the pointer of an analogic meter and contour analysis to extrapolate the meter scale. Fang et al. [6] adopted a faster method to identify the pointer angle by using a least squares method. Chi et al. [7] implemented a more robust algorithm to locate the gauge display using the region growing method. Belan et al. [8] proposed a new solution to detect the pointer without the need of segmenting/binarizing the image, based on the Bresenham algorithm [9].

### Motivations and Contributions

Those algorithms work very well for inspections routines. The autonomous agent knows where the gauges are located and knows their main features (scale values and unit of measurements), therefore it can be programmed to obtain a frontal view of the gauge and to detect just the needle angle. However, in the case of critical dysfunctions or unforeseen emergency situations, it is useful to have autonomous agents capable of dealing with *unstructured environments*, where no prior knowledge is available, and equipped with more robust end-to-end algorithms to obtain situational-awareness and to secure the infrastructure. In a scenario where an explosion occurred in a sector of a plant, a robot could be deployed to search for pressure gauges and read and evaluate if eventual values also represent an imminent danger in other sectors of the plant. In this context, it may be impossible to obtain a front view of the gauge display, and the gauge scale is unknown. Therefore, most gauge-reading algorithms would fail, as they miss the following features:

- Gauge detection from scene.
- Display rectification.
- Gauge scale reading.
- Near real-time function.

Some recent advances in this application encompass some of the points that are listed above. The algorithm developed by Dumberger et al. [10] goes in that direction, implementing gauge detection, Optical Character Recognition (OCR) for scale reading and near real-time operation (2 s per reading). An advanced algorithm is also shown by Li et al. [11], where they apply a neural network to detect and read the text on the gauge display and use this information to rectify the perspective of the gauge and reconstruct the scale. The existing solutions here discussed are summarized in Table 1, where we list their features.

**Figure 1:**
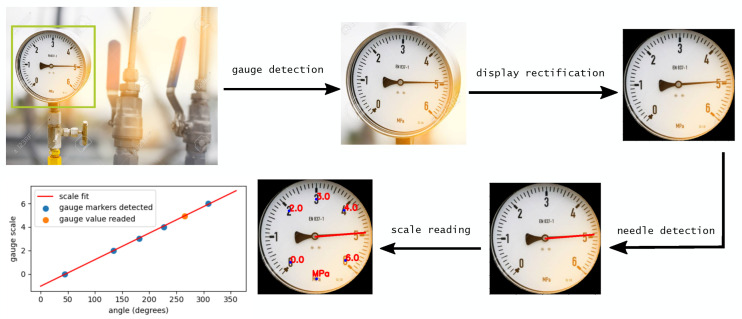
GAUREAD algorithm’s steps: gauge detection from scene, display rectification from a perspective angle, needle/pointer detection and scale reading and reconstruction.

From Table 1, it is possible to observe that it is still missing a full end-to-end algorithm that includes all the necessary features to ensure an autonomous reading in unstructured conditions. In [10], the display rectification step is missing, which means that the method does not work with gauges that have a perspective angle. In [11], the rectification is implemented, but there is no method to detect the presence of the gauge from the scene. Moreover, the scale reconstruction is limited to the peculiar type of gauge analysed, as there is no OCR performed. With this paper, we aim at filling this gap, presenting an algorithm that comprises all necessary steps to read a gauge without any previous knowledge. In the first section of this paper, we present the steps of the algorithm (named GAUREAD: GAUge READer), describing the four main stages: gauge detection, display rectification, needle detection and scale reading (Figure 1). In the subsequent sections, we report on a series of experiments to characterize the accuracy of GAUREAD and comment on the results obtained. Finally, conclusions and future perspectives are discussed in the last section.

## 2. GAUREAD

### 2.1. Gauge Detection

The input of the algorithm can be an image file or a video frame. Similarly to [10], we used a deep neural network of the YOLO [12] family (YOLOv4-tiny) to perform gauge detection from the scene. The bounding box output of the detection algorithm is used to crop the gauge and pass the new image to the subsequent step of the algorithm.

### 2.2. Display Rectification

**Figure 2:**
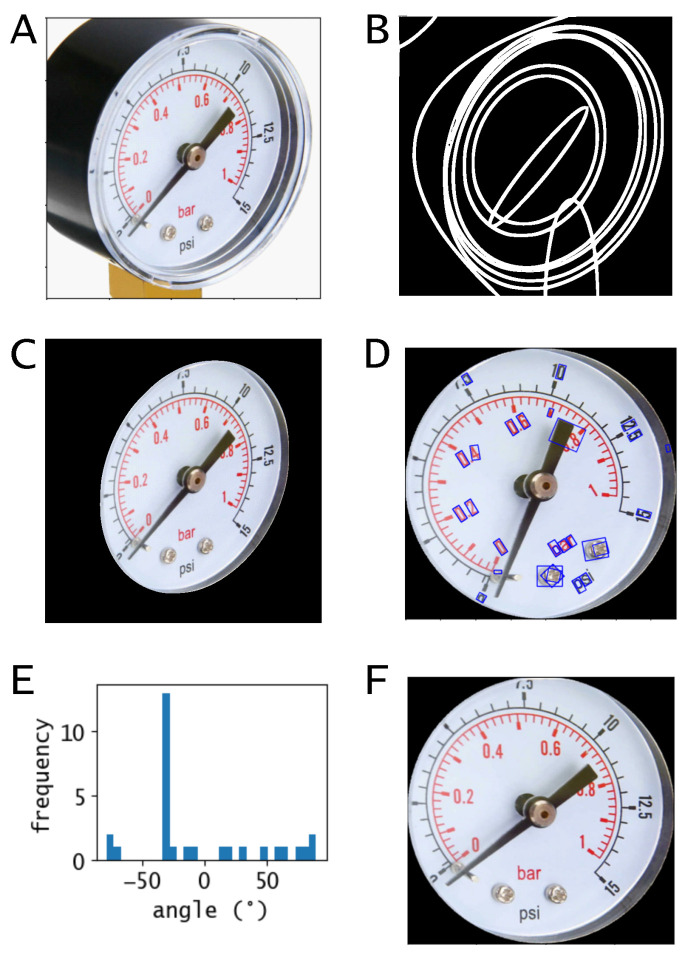
Display rectification algorithm. (**A**) Analogue gauge with from a perspective view. (**B**) Ellipse fitting of the ten largest contours. (**C**) Display segmentation. (**D**) Display rectification and text detection. (**E**) Distribution of the inclination angles of the detected text. (**F**) Rotation to the text reading perspective.

In this work, we consider gauges that have a circular shape, as those are the most spread in industrial facilities. As mentioned in the introduction, most gauge-reader algorithms follow this assumption and use Circle Hough Transform to detect and segment the gauge display. This circle is used to generate a polar reference system that can map the gauge values to the polar angle coordinates. However, if the gauge is tilted or the camera has a view angle (like the gauge depicted in Figure 2A), the display has an elliptical shape and the Hough Transform cannot be used. Moreover, the display needs to be straight in order for the text to be read and the gauge scale to be reconstructed. Contrary to [11], where the authors use only the text location to rectify the display as the gauge is not circular, here we propose a geometrical approach that comprises the following steps:

- Display ellipse contour detection.
- Ellipse to circle transformation.
- Circle rotation for text alignment.

The first step consists of extracting the 10 largest contours of the binarized gauge image. An ellipse is fitted to each contour using the pre-built OpenCV function fitEllipse. Among these 10 ellipses, there is the display as well as other features like the gauge-case, the needle and other eventual contours (Figure 2B). In order to choose the correct ellipse, that is the one corresponding to the display contour, three parameters are calculated for each ellipse. A skew factor $S_{f}$, an area factor $A_{f}$ and a central factor $C_{f}$, defined as follow:

(2) $$S_{f}=\frac{\left|a-b\right|}{a+b}$$

(3) $$A_{f}=\frac{ab}{wh}$$

(4) $$C_{f}=\sqrt{{\left(x_{e}-x_{c}\right)}^{2}+{\left(y_{e}-y_{c}\right)}^{2}}$$

where a,b are the ellipse width and height, $x_{e},y_{e}$ the ellipse centre, w,h the image width and height and $x_{c},y_{c}$ the image centre (all dimensions in pixel). The three parameters are used to sort the 10 ellipses since the one corresponding to the display is likely not very skewed, not far from the centre of the image and not small compared to the image area. We selected the acceptance intervals as $S_{f}<0.4$, $C_{f}<0.2$ and $0.4<A_{f}<0.8$ through a heuristic procedure. Among the ellipses that satisfy those criteria, the one with the smallest area is selected (Figure 2C). The display contour is then rotated to vertically align the long axis of the ellipse and inscribed in a rectangle used to crop the gauge image. The new image is transformed into a square so that the display has finally a circular shape.

At this stage, the display has still an unpredictable orientation and needs to be rotated in order to make the text readable for the OCR engine. The rotation angle is calculated by estimating the text orientation on the display. Additionally, here we adopt a geometrical approach in contrast to the deep learning adopted by Li et al. [11], to achieve faster computation time. Contours below an area threshold ($max(A_{c})/50$) are inscribed in rectangles (Figure 2D), where $A_{c}$ is the ensemble of the areas of the ten largest contours. The peak of the histogram distribution of the orientation angles of the rectangles is selected as rotation angle (Figure 2E).

### 2.3. Needle Detection

**Figure 3:**
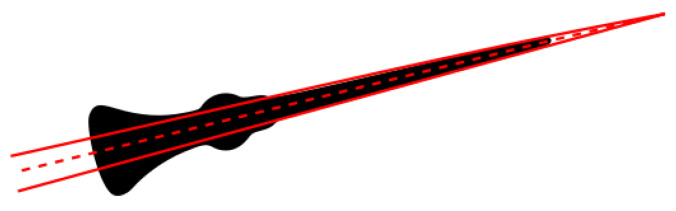
Needle angle estimation. Hough Linear Transform to detect the needle profiles (red continuous lines) and average line for the inclination angle (red dashed line).

Once the display is rectified (Figure 2F), the needle is detected using Linear Hough Transform, as the standard in gauge reader algorithms [5]. However, while in those algorithms, the lines detected on the needle are averaged to get the centre-line, in our algorithm, we select the needle angle as the polar angle of the intersection between the lines following the needle edges. In this way, the needle angle cannot be mistaken for the opposite direction of the needle centre-line (Figure 3). This approach is valid as long as the needle has a slender triangular shape, which is the typical case for all general types of needles and pointers.

### 2.4. Scale Reading

At this stage of the algorithm, the polar representation of the display and the needle angle are calculated; therefore, the gauge value could be obtained using Equation (1) if the gauge scale was known. However, as we argued in the introduction, there are applications where the gauge scale is not previously determined and needs to be reconstructed. The objective therefore is to read the text representing the scale digits and the unit of measurements. We developed a scale reading algorithm that operates in three steps: text detection, text recognition and scale reconstruction.

Text detection is again performed through contour analysis, as described in the paragraph on display rectification. Bounding boxes of single characters belonging to the digit or unit of measurements are identified through a maximum distance threshold and merged together to obtain the bounding boxes of the whole digit/unit. The centre of the bounding box is used to represent the text location in polar coordinates.

**Figure 4:**
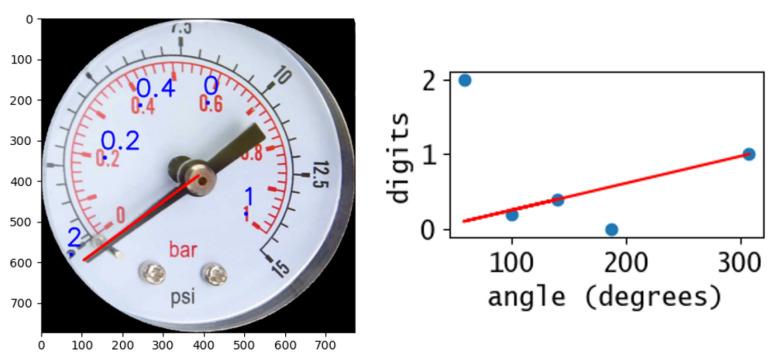
Scale reconstruction. OCR engine output on the gauge display and scale interpolation of the digits’ polar angle coordinates.

Text recognition is performed using Tesseract v5 OCR engine [13]. The engine runs through every subfigure enclosed in the bounding box, so that each output string is mapped to a polar coordinate. In the string list, there are the digits displayed on the gauge, the units of measurements (if the gauge has two scales, for example in bar and psi), other text like gauge model number and brand, and likely many random text strings belonging to false text detection in the first step. Moreover, many characters can be easily misinterpreted, leading to the wrong value of real text detected (Figure 4). Therefore a scale reconstruction algorithm is necessary to select the strings of real interest. As done in [10], all strings are compared to a list of possible units of measurements to match the correct scale unit.

Regarding the actual numerical reconstruction of the scale, a new approach is developed. All strings containing a numerical value ($v_{i}$) are plotted as function of the related polar angle ($\theta_{i}$). Since we assume that the gauge scale is linear, the values that are part of this scale lay on the same line. The line represents the scale linear function, which has to be determined to calculate the gauge value (Figure 4). To first approximate the linear function, we compute the slope of the line passing through each couple of points. Since the values that do not belong to the gauge scale are randomly scattered, the slope of the linear function (*m*) corresponds to the peak of the histogram of the slope distribution. The constant term of the linear function (*q*) is found analogously, by calculating it for all the parallel line passing through each point. This linear function could be used to determine the gauge value, but it is not optimal due to inevitable numerical error caused by the histogram binning. Therefore, the best practice is to use this first approximation of the linear function to filter out all the values that have a bad fit, and perform a linear regression of the remaining values. The fit accuracy is calculated as the coefficient of determination of the single data point (Equation (5)), which computes the normalized squared residuals. A residual equal to 0 is a perfect fit and corresponds to an accuracy equal to 1. Here, we use 0.95 as threshold to discard values. Studies suggest that the coefficient of determination is the most informative metric to evaluate a linear regression model [14]. Here, $\bar{v}$ is the mean of all values.

(5) $$acc=1-{\left[\frac{v_{i}-m\theta_{i}-q}{v_{i}-\bar{v}}\right]}^{2}$$

The remaining values are linearly interpolated to obtain the final scale linear function, and the gauge value is calculated in function of the polar angle of the needle position. As one main assumption is that the gauge scale is linear, it is enough to precisely locate at least two digits represented on the display to be able to reconstruct the scale and read the gauge. This makes it possible to read gauges where parts of the display are not interpretable, due to unwanted reflections or dirt on the display glass, as long as the needle position is clearly visible as well as two digits on the display.

## 3. Experiments

**Figure 5:**
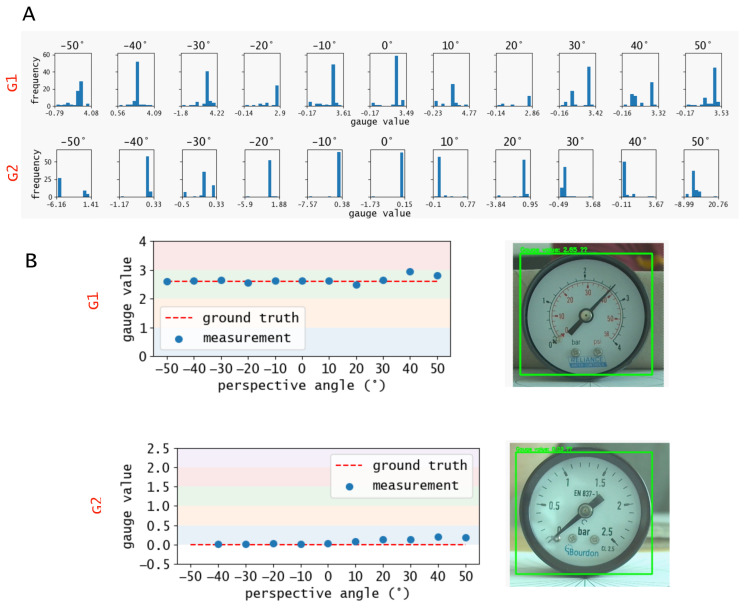
Experimental results. (**A**) Histograms of the measurements for each perspective angle for the tests on gauges G1 and G2. (**B**) Measurements, selected as the peaks of the histograms of subfigure (**A**), against the ground truth for the two test. The test conducted at −50° for the gauge G2 did not provide a reliable result.

In order to assess the accuracy of GAUREAD, we perform an experimental validation. We test our algorithm on the two pressure gauges depicted in Figure 5. We refer to the them as G1 and G2 during the text. In each test, the gauge is set to a value and placed in front of the camera with a perspective angle varying between −50° and 50°. An acquisition is run every 10° step of the perspective angle and consists of a real-time video stream at 1 FPS, enough for GAUREAD to perform its analysis, for 60 s. Since G1 displays two scales (psi and bar), the values measured in psi are manually converted in bar to be consistent. The gauge-reader algorithm is implemented in python on the NVIDIA Jetson Nano 4GB equipped with a Raspberry HQ camera (12.3 megapixel Sony IMX477 sensor) through the MIPI CSI-2 interface. The Jetson Nano features a 128-core Maxwell GPU that makes it very suitable to run neural networks for object detection. Given the small dimensions (69 mm × 45 mm), this setup can be easily installed on any mobile robot to perform autonomous operations. In the previous section, we mentioned that the gauge detection is performed using a YOLO deep neural network. We opted for YOLOv4-tiny [15] implemented in the Darknet framework due to his faster inference speed on the Jetson Nano (16–17 FPS). YOLOv4-tiny is the compressed version of YOLOv4 designed to be used on machines with low computing power. Its average precision is of the order of 40 percent in Average Precision at Intersection over Union (IoU) equal to 0.5 (AP50), whereas YOLOv4 reaches 65% [16]. This lower precision, however, is compensated by the fast inference speed. Here, we adopted YOLOv4-tiny in inference mode as we did not train the model, but used the pre-trained model based on the COCO dataset. We found that the COCO class *clock* can be used to accurately detect pressure gauges. In Table 2, we report the average and standard deviation of the Confidence Score (CS, defined as the probability that the box contains the object of the specific class multiplied by IoU) obtained with YOLOv4-tiny when detecting the two different gauges G1 and G2 for the different perspective angles considered in our experiments. Except for the gauge G2 at −50°, where indeed GAUREAD failed to output a measurement as shown in the next section, the average CS is always above 0.75, showing the feasibility of this method to detect gauges from scenes. Moreover, the pre-trained model may enable additional applications given the higher number of classes available. Display rectification and needle detection are also good in terms of time consumption as they require about 50–80 ms. On the other hand, the scale reading stage is the time bottleneck since the OCR engine requires the highest amount of time. Using the wrapper Pytessy [17] to interface with Tesseract OCR engine, we were able to speed up to 0.6 s per frame. A single frame goes trough the algorithm in 0.8 s in average, where 75% of the time is consumed by OCR text recognition.

## 4. Results and Discussion

**Figure 6:**
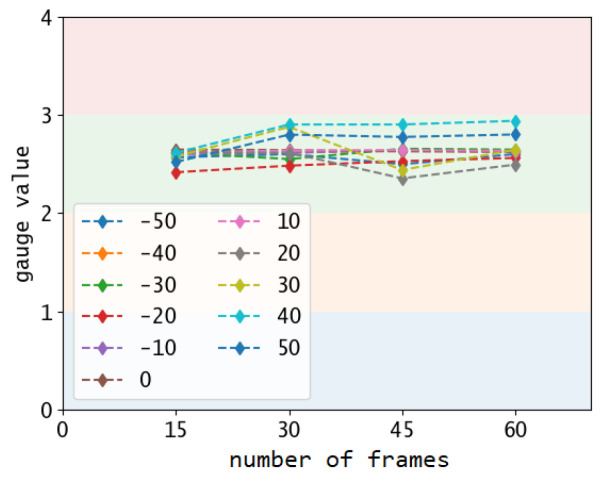
Measured values for increased amount of frames sampled for the gauge test G1.

The raw data is the ensemble of the values measured for each frame during the acquisition time. Since the algorithm is not always able to provide a value, the size of the ensembles can vary between the tests even if the acquisition time is fixed. The histogram distribution of the ensembles is reported in Figure 5A for each test. The objective is to determine the estimated gauge value from the histogram. All distributions are characterised by a clear peak in frequency and a wide distribution of scattered values. In this context, the mean value is not a significant metric since the relative standard deviation of the ensemble is very large and not symmetric, which means that there is no systematic error in the measurements, but rather an aleatory error. In the tests, the relative standard deviation varies between 15–50%. On average, roughly 30% of the values are completely out-of-scale (not in the range of the gauge scale) due to a errors in numbers recognition. In fact, the OCR engine minimal variations in the image correspond to large misinterpretation of the display’s text. This is clear by computing only the needle angles, before performing the scale conversion. In this case, the relative standard deviations are much lower, varying from a minimum of 0.5% to a maximum of 5% for high perspective angles. However, since the estimated value is systematically consistent and the other values are randomly scattered, the histogram peak is an accurate metric and does not vary significantly from the ground truth, as depicted in Figure 5B. Therefore, we identify the histogram peak as measured value. The out-of-scale values can have an impact only if a small amount of frames is analysed and, as depicted in Figure 6, already after 15 frames the measurement converges to the in-scale value.

It is interesting to note how the measurements tend to diverge from the ground truth for higher perspective angles, due to the higher distortion. This trend is consistent with the analysis reported by Li et al. [11], where the authors also studied the impact of perspective angles on their gauge reader. Consistently with [11], we define a measurement error as the difference between the measured value and the ground truth, considered as the manual image analysis of the gauge with a zero angle perspective, normalized by the full-scale value of the gauge. The error values are depicted in Table 3, and expressed in percentages. Errors exceeding 5% occur at high perspective angles, where the distortion of the frame is more significant. The test conducted at −50° for the gauge G2 did not provide a meaningful measurement.

In [11], the authors reported a measurement error of less than 2%. However, their method does not include the OCR scale reading, which is the main source of error in GAUREAD as mentioned in the previous paragraphs. Therefore, to have a reliable comparison, we compute our measurement error again before the scale reading step, so that only the display rectification is considered. This means that the error is computed for the estimated needle angle. In this case, the measurement errors drop down to less than 3%, showing performances of display rectification comparable with the state of the art. Moreover, to show the benefits of the rectification step, we compared our display detection performances against the Circle Hough Transform (CHT) for the same dataset from the test G1. The comparison is reported in Table 4, where we compute the measurement error of the needle angle. CHT method failed at detecting the gauge display at perspective angles higher than 30°, since the gauge display has an elliptic shape due to the perspective distortion, proving the necessity of display rectification methods as the one developed in this paper. At lower perspective angles, CHT and GAUREAD have similar performances as the gauge is well fitted by the circular shape, confirming the reliability of GAUREAD. Despite not using a dedicated neural network to identify and read the text on the gauge display, we achieved a comparable performance with [11].

## 5. Conclusions and Future Work

In this paper, we presented GAUREAD, an algorithm that automatises analogue gauge reading in real-time and without any prior knowledge of the gauge. We developed and presented the complete algorithm, from gauge detection and display rectification, to display reading and scale reconstruction. In such a way, the analogue gauge can be read without any prior information regarding its position on the scene, its orientation and its scale. At this current stage, our solution is currently limited to gauges that have a circular shape. Regarding accuracy and speed performances, the main limitation of our system is the Tesseract OCR engine for reading the gauge display. This step of the algorithm is the most time consuming (75% of the total time) and is prone to uncertainties and inaccuracies. A collection of multiple frames was needed to establish the correct gauge value and eliminate the false measurements caused by the OCR misreadings. As future work, the OCR engine could be replaced by a learning algorithm that is specifically trained to detect the scale numbers on any gauge display. This could potentially lead to a faster speed and improve the accuracy and the overall FPS performance of GAUREAD.

## Figures and Tables

**Table 1:** Comparison of existing gauge reader’s methods and their features.

First Author	Gauge Detection	Display Rectification	Calibration	Scale Reading
Alegria [5]	×	×	✓	×
Fang [6]	×	×	✓	×
Chi [7]	×	×	✓	×
Belan [8]	×	×	✓	×
Li [11]	×	✓	✓	✓
Dumberger [10]	✓	×	✓	✓

**Table 2:** Mean and standard deviation (STD) of the confidence score (CS) obtained by YOLOv4-tiny in detecting the two gauges considered in our experiments.

Angle (°)	Mean CS G1	STD CS G1	Mean CS G2	STD CS G2
−50	0.89	0.04	0.46	0.12
−40	0.89	0.05	0.78	0.05
−30	0.92	0.03	0.84	0.03
−20	0.89	0.02	0.90	0.01
−10	0.90	0.03	0.88	0.02
0	0.90	0.02	0.79	0.05
10	0.95	0.01	0.77	0.03
20	0.91	0.03	0.88	0.02
30	0.92	0.02	0.94	0.01
40	0.83	0.05	0.86	0.05
50	0.84	0.07	0.92	0.03

**Table 3:** Measurement error on the gauge value for the two different gauges G1 and G2.

Angle (°)	Error G1 (%)	Error G2 (%)
−50	0.05	-
−40	0.6	0.84
−30	1.15	0.84
−20	0.95	1.12
−10	0.55	0.8
0	0.65	1.4
10	0.85	3.56
20	2.65	5.36
30	1.15	5.64
40	8.5	7.92
50	5	7.56

**Table 4:** Error’s comparison on the estimated needle angle using GAUREAD vs. CHT (Circle Hough Transform) for test G1 and errors reported in Ref. [11] for their AC ammeter test.

Angle (°)	Error GAUREAD (%)	Error Ref. [11] (%)	Error CHT (%)
−50	0.5	1.62	-
−40	1.45	1.20	-
−30	0.95	0.78	2.07
−20	0.71	0.51	0.41
−10	0.73	0.29	0.46
0	0.06	0.16	0.02
10	2.61	0.35	1.09
20	0.76	0.57	1.65
30	2.44	0.89	2.05
40	3.12	1.29	-
50	2.75	1.69	-

## Data Availability

The datasets generated during and/or analysed during the current study are available from the corresponding author on reasonable request.

## Abbreviations

The following abbreviations are used in this manuscript:

GAUREAD	GAUge READer
OCR	Optical Character Recognition
FPS	Frame Per Second
CS	Confidence Score
STD	Standard Deviation
